# Surgical mask on top of high-flow nasal cannula improves oxygenation in critically ill COVID-19 patients with hypoxemic respiratory failure

**DOI:** 10.1186/s13613-020-00744-x

**Published:** 2020-09-29

**Authors:** Virginie Montiel, Arnaud Robert, Annie Robert, Anas Nabaoui, Tourneux Marie, Natalia Morales Mestre, Maerckx Guillaume, Pierre-François Laterre, Xavier Wittebole

**Affiliations:** 1 Intensive Care Unit, Cliniques Universitaires Saint‐Luc, UCLouvain, 10 avenue Hippocrate, 1200 Brussels, Belgium; 2 Institut de Recherche Expérimentale et Clinique, Pôle Epidémiologie et Biostatistique, UCLouvain, Belgium; 3 Intensive Care Unit, Cliniques Universitaires Saint‐Luc and Institut de Recherche Expérimentale et Cliniques (IREC), Pôle de Pneumologie ORL et Dermatologie (PNEU), UCLouvain, Belgium

**Keywords:** Hypoxemic failure, High-flow nasal cannula, COVID-19 patient

## Abstract

**Objective:**

Critically ill patients admitted in ICU because of COVID-19 infection display severe hypoxemic respiratory failure. The Surviving Sepsis Campaign recommends oxygenation through high-flow nasal cannula over non-invasive ventilation. The primary outcome of our study was to evaluate the effect of the addition of a surgical mask on a high-flow nasal cannula system on oxygenation parameters in hypoxemic COVID-19 patients admitted in ICU who do not require urgent intubation. The secondary outcomes were relevant changes in PaCO_2_ associated with clinical modifications and patient’s feelings.

**Design:**

We prospectively assessed 21 patients admitted in our mixed Intensive Care Unit of the Cliniques Universitaires Saint Luc.

**Main results:**

While FiO2 was unchanged, we demonstrate a significant increase of PaO_2_ (from 59 (± 6), to 79 mmHg (± 16), *p* < 0.001), PaO_2_/FiO_2_ from 83 (± 22), to 111 (± 38), *p* < 0.001) and SaO_2_ (from 91% (± 1.5), to 94% (± 1.6), *p* < 0.001), while the patients were under the surgical mask. The SpO_2_ returned to pre-treatment values when the surgical mask was removed confirming the effect of the device rather than a spontaneous positive evolution.

**Conclusion:**

A surgical mask placed on patient’s face already treated by a High-flow nasal cannula device improves COVID-19 patient’s oxygenation admitted in Intensive Care Unit for severe hypoxemic respiratory failure without any clinically relevant side.

## Background

A novel coronavirus named severe acute respiratory syndrome coronavirus 2 (SARS-CoV-2) is responsible for an acute and a rapidly evolving illness that the World Health Organization termed Coronavirus Disease 2019 (COVID-19) [1]. This disease spread around the entire globe because of its great contagiousness and led to a severe public health problem. While most patients present with mild respiratory symptoms, some present with severe pneumonia requiring hospitalization and in the most severe cases Intensive Care Unit (ICU) admission.

This situation induced an increased pressure on ICUs that are stretched beyond their capacity to provide specific care for the most severely ill patients including the need of specific materials for mechanical ventilation such as ventilators [2].

Actually, hypoxic respiratory failure is observed in 14% of patients that will require hospitalisation with supplemental oxygen administration. Furthermore, a severe acute respiratory failure is observed in 5% of these patients despite conventional oxygen therapy, requiring hospitalisation in ICU [3]. This COVID-19 associated pneumonia is well characterized by a bilateral infiltrates on chest X-ray and by bilateral ground-glass opacifications with occasional consolidation in multi-lobe lesions on chest CT [4].

In this critical pandemic situation, where the incidence of COVID-19 severe patients requiring specific intensive treatment continues to rise with limited resources such as ventilators, we tried to improve oxygenation parameters of our patients with easy-to-perform procedures. Our ICU used high-flow nasal cannula (HFNC), as recommended by the guidelines for acute hypoxemic respiratory failure in COVID-19 adults patients that do not require urgent endotracheal intubation [5]. In our hospital, to decrease the risk of viral transmission from exhaled air, a surgical mask is actually recommended on patient’s face during HFNC treatment when healthcare workers are in the room. Indeed, recent data suggest a variable increase in the mean distance of droplet dispersion from coughing with the use of HFNC devices, especially when the flow rate is increased [6, 7].

The primary outcome of our study was to evaluate the effect of the addition of a surgical mask on an HFNC system on oxygenation parameters (PaO_2_) in hypoxemic COVID-19 patients admitted in ICU who do not require urgent intubation. The secondary outcomes were relevant changes in PaCO_2_ associated with clinical modifications and patient’s feelings.

## Materials and methods

This study is a prospective monocenter study performed in a 22-bed mixed ICU at the Cliniques Universitaires Saint Luc. We enrolled all consecutive COVID-19-infected patients admitted in ICU who did not require urgent intubation. This study was performed only when the investigators were present at the time of admission. All patients were connected to a HFNC device (Optiflow TM RT202, Fisher & Paykel, Auckland, New Zealand) upon ICU admission and were monitored through our usual system (Intellivue Patient Monitor MP70, Philips Medizin Systeme Boeblingen GmbH, Boeblingen, Germany).

Inclusion criteria included hypoxemic and awake patients requiring HFNC who were relatively stable under this treatment and without a presumed intubation or anticipated changes to respiratory clinical management within the next 2 h. Exclusion criteria of this study assimilate exclusion criteria for classical HFNC including an altered consciousness, confusion, persistent hypoxemia or hypoventilation, respiratory acidosis, exacerbation of asthma or chronic respiratory failure, cardiogenic pulmonary oedema, circulatory failure with use of vasopressor, the need for non-invasive or invasive mechanical ventilation [8]. During the study, the patient’s feeling was evaluate by asking questions about eventual sensation of suffocation or the onset of dyspnoea and by analysing the behaviour’s patient by the investigators that monitored the experiment.

The enrollment process allowed us to include 21 patients between 27/03/2020 and 22/04/2020. All the patients agreed to participate in the study and had supplementary arterial blood gas drawn through the arterial line since the surgical mask on the HFNC device was a special request of our hospital (hospital hygiene department) for all patients admitted in ICU. Written informed consent was obtained from all participants before inclusion. We excluded three patients that required immediate intubation upon their arrival in ICU and one patient admitted during the night required HFNC but with a very rapid clinical improvement in the next morning that no longer justified administration of an HFNC device.

## Statistical analysis

Because no preliminary data were available for sample size determination, we made some hypotheses, based on our clinical knowledge. Assuming a standard deviation of 15 mmHg in the response of matched PaO_2_ measurements, a sample size of at least 20 patients allows having a power of 80% to detect a mean increase of at least 10 mmHg in PaO_2_, at the statistical significance level of 0.05. Such assumption corresponds to a Cohen’s effect size of 0.67; the larger the effect size the lower the sample size.

All results are expressed as mean ± SD. Data with and without surgical mask were compared using paired t tests, after checking with Q–Q plots if differences were normally distributed. Values with repeated measures like the SpO_2_ were analysed using a within-factor ANOVA with F test and a Bonferroni correction was used for multiple comparisons. All tests were two sided and a *p* < 0.05 was considered as statistically significant. Analyses were performed with Graph Pad Prism version 8.1.2.

## Protocol

Subjects were enrolled a few hours after admission in our ICU, while lying in a calm environment in a single room. Placement of an inline arterial catheter was performed as usual upon ICU admission. Oxygenation through the HFNC device was started and adapted in all patients with a SpO_2_ target above 90%. The flow was fixed at 60 L/min. The temperature was set at 31–37 °C. These parameters were then fixed without any modification during the ongoing experiment unless deleterious evolution was observed. In particular, we did not change the FiO_2_ parameters during the experiment. Patients were placed in a semi-recumbent position, with the HFNC already in place.

A first blood gas measurement after a minimum of 30 min of HFNC was performed. Then, a surgical mask was set on the patient’s face, encompassing the nose to the chin. The mask was placed by the study investigator to avoid any misplacement and to assure the absence of leak. Correct placement of the mask was assessed all along the procedure. A second blood gas measurement after a minimum of 30 min of HFNC with the surgical mask was performed. This blood gas measurement was performed as standard of care to verify the absence of secondary side effects including hypercapnia. The surgical mask was then removed and SpO_2_ was registered after another 30 min of HFNC only.

Recorded haemodynamic and respiratory parameters on each study phase included heart rate, blood pressure, SpO_2_ and respiratory rate.

## Results

Our patient population consisted of 21 patients (18 males and 3 females). The mean age was 60 years (± 12.3) and the mean body mass index was 28 kg/m^2^ (± 4). The mean FiO_2_ administration through HFNC at the beginning of the study protocol was 75% (± 18%) and they all were receiving oxygen at a flow rate of 60 L/min. The maximum SOFA score of our patient population was 5 with a range of 3–6, mainly due to the severe hypoxemia. All our patients presented a normal neurologic evaluation (Glasgow Coma Scale of 15/15). The characteristics of the patients are detailed in Table 1.

**Table 1:** Characteristics of the patients

Patient	Age (years)	Sex	Body max index (kg/m^2^)	FiO_2_	SOFA score at admission	Glascow Coma Scale
1	85	M	28	0.6	5	15
2	52	M	34.2	0.8	4	15
3	54	M	23.8	1	4	15
4	37	M	23.3	0.5	3	15
5	67	M	33.4	0.75	4	15
6	69	M	22.5	0.4	5	15
7	50	M	31.2	0.5	3	15
8	62	F	35.6	0.9	4	15
9	60	M	26.2	1	4	15
10	57	M	27.3	1	4	15
11	56	M	27.8	0.7	6	15
12	73	M	24.2	0.6	4	15
13	64	M	33.9	1	4	15
14	76	M	26.3	0.6	5	15
15	76	M	23.4	0.8	4	15
16	50	M	28.7	0.7	5	15
17	51	F	23.8	0.8	4	15
18	46	M	30.8	0.9	4	15
19	54	M	31.6	0.6	4	15
20	81	F	22.9	0.75	4	15
21	47	M	31	0.8	4	15
Mean ± SD	60 ± 12	M: 86%	28.24 ± 4.11	0.75 ± 0.18	(3; 6)	15

*SOFA* Sequential organ failure assessment

**Fig. 1:**
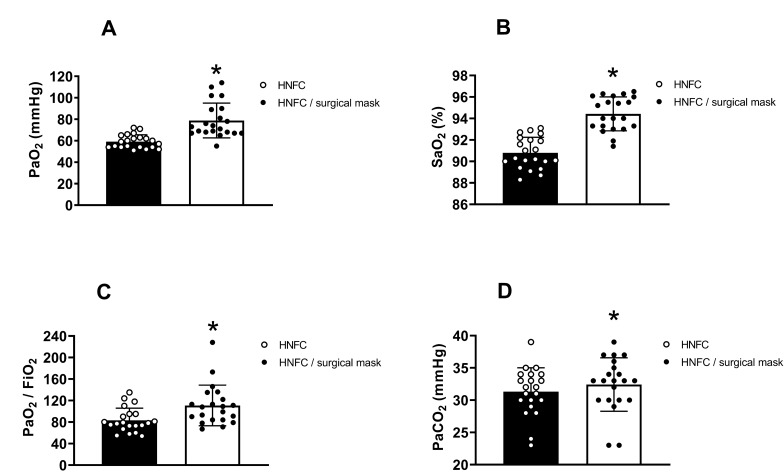
Respiratory parameters with high-flow nasal cannula (HFNC) alone and in the presence of a surgical mask (HFNC/surgical mask). These data showed an improvement in all variables. *p < 0.05 compared to HNCF alone in paired *t* tests

We did not observe any significant change in haemodynamic parameters during all the process, while we noticed a small decrease of the respiratory rate with the surgical mask set on the HFNC device (from a rate to 27.8/min (± 5.5) to 26.2/min (± 5.6), *p* < 0.05). We observed a significant improvement of the PaO_2_ from (59 mmHg (± 6), to 79 mmHg (± 16), *p* < 0.001) (Fig. 1a), the SaO_2_ (from 91% (± 1.5), to 94% (± 1.6), *p* < 0.001) (Fig. 1b), the PaO_2_/FiO_2_ (from 83 (± 22) to 111 (± 38), *p* < 0.001) (Fig. 1c). We also observed a change in the mean PaCO_2_ (from 31 mmHg (± 3) to 32 mmHg (± 4), *p* < 0.002) (Fig. 1d). All these parameters are detailed in Table 2 for each patient.

**Table 2:** Haemodynamic and respiratory parameters of all patients (from 1 to 21)

Phase 1: High-flow nasal cannula							Phase 2: High-flow nasal cannula + surgical mask							Increase from phase 1 to 2 (%)	
Blood pressure (BP) systolic/mean/diastolic (mmHg)	Heart rate (min)	Respiratory rate (min)	PCO_2_ (mmHg)	PaO_2_ (mmHg)	SaO_2_ (%)	PaO_2_/FiO_2_ ratio	Blood pressure (BP) systolic/mean/diastolic (mmHg))	Heart rate (min)	Respiratory rate (min)	PCO_2_ (mmHg)	PaO_2_ (mmHg)	SaO_2_ (%)	PaO_2_/FiO_2_ ratio	PaO_2_/FiO_2_ ratio PaO_2_	SaO_2_
121/78/56	81	32	24	66	92	110	124/79/56	76	31	23	68	93	113	3	1
109/76/58	82	40	35	65	91.6	81	105/77/60	85	32	34	74	94	93	14	3
168/99/67	91	27	32	67	92.7	67	157/98/75	88	26	33	90	96.3	90	34	4
177/105/76	93	33	39	62	90,1	124	168/108/85	92	31	39	114	96.3	228	84	7
137/103/89	88	21	33	71	92.9	95	136/86/61	87	21	33	102	96	136	44	3
135/91/63	70	18	34	54	90	135	117/78/55	70	18	37	69	93.3	173	28	4
123/93/78	95	25	30	59	92.6	118	143/110/90	85	22	32	73	95.2	146	24	3
130/72/46	78	31	32	52	89.4	58	135/81/56	82	28	33	110	96.1	122	112	7
110/84/68	98	28	30	55	89.3	55	107/83/67	97	26	30	67	94	67	21	5
101/64/53	69	25	34	54	89.1	54	115/61/39	71	22	36	71	93.3	71	31	5
134/85/69	100	30	30	54	88.3	77	130/83/65	102	30	30	76	94	109	41	6
120/74/53	80	28	28	57	91.9	95	130/80/62	82	27	29	81	96.1	135	42	5
133/85/69	110	31	29	60	93.1	60	135/91/72	109	30	30	78	96.5	78	30	4
128/80/67	81	28	31	51	90.1	73	122/73/60	84	24	3	55	91.4	79	8	1
128/86/63	84	24	35	62	90	78	132/88/65	86	23	35	67	93.3	84	8	4
126/84/58	105	39	23	52	90.2	74	114/80/60	94	43	23	69	95.5	99	33	6
147/92/65	94	29	33	60	91	75	146/89/62	94	30	37	67	93	84	12	2
135/94/66	103	30	34	72	92.2	80	133/92/64	99	29	37	102	95.5	113	42	4
102/75/68	70	20	33	54	90.3	90	119/84/57	69	20	33	65	92.8	108	20	4
140/95/68	125	24	28	55	88.7	73	130/91/68	130	23	30	68	91.9	91	24	4
129/85/65	110	25	31	63	91.1	79	125/84/63	105	26	34	89	95.4	111	41	7
Mean ± SD															
Systolic BP 130 ± 18 Mean BP 85 ± 10 Diastolic BP 65 ± 9	90 ± 14	28 ± 5	31 ± 1	59 ± 1	90.8 ± 1	83 ± 22	Systolic 130 ± 15 Mean BP 85 ± 11 Diastolic BP 64 ± 11	89 ± 14	26 ± 5	32 ± 1	79 ± 3	94.4 ± 1	110 ± 37	33.1 ± 25	4 ± 1.7

**Fig. 2:**
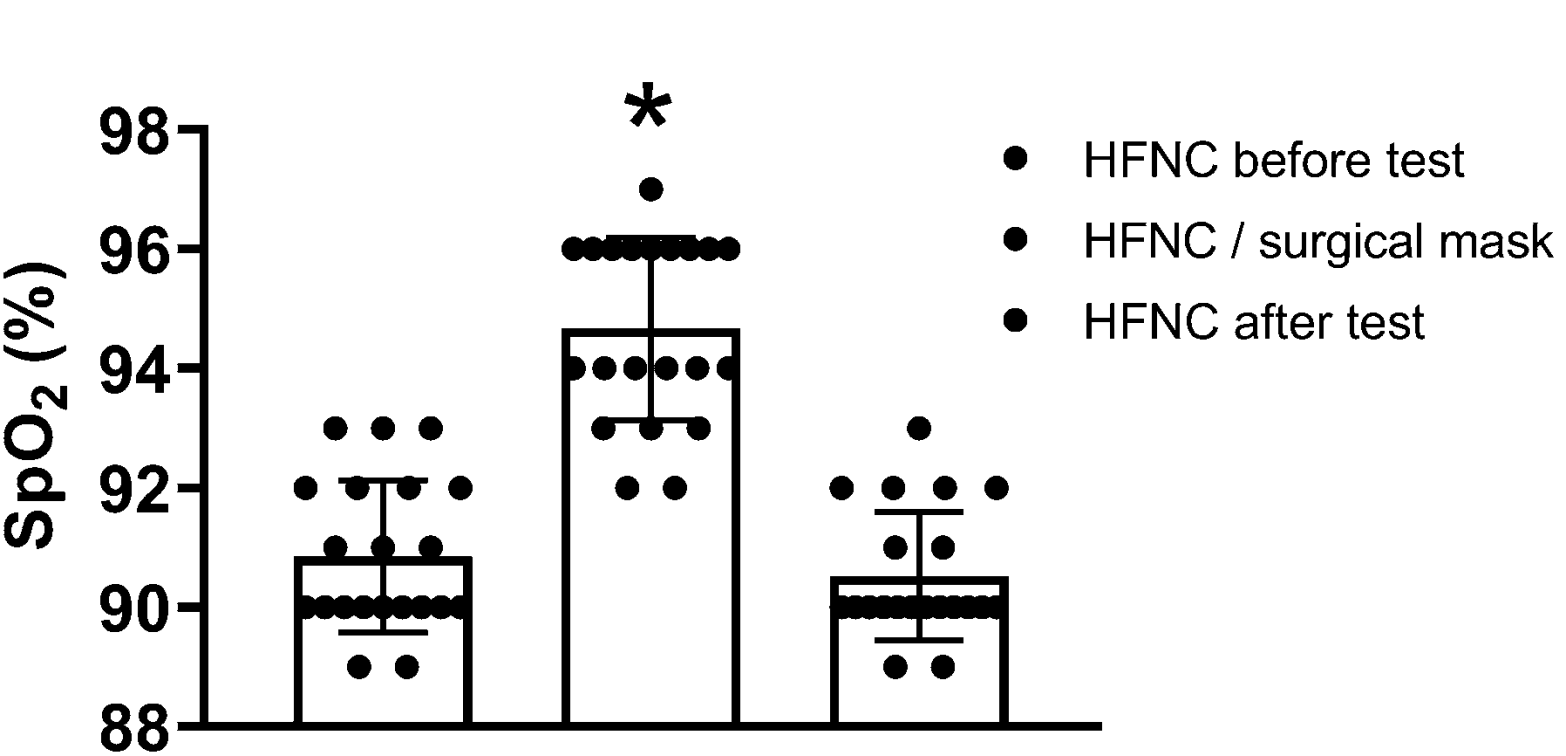
Repeated SpO_2_ performed with high flow nasal cannula before the test alone (HFNC before test), in presence of the surgical mask (HFNC/surgical mask) and finally again with high flow nasal cannula alone (HFNC after test). *p < 0.05 in a within-factor ANOVA with Bonferroni correction for multiple comparisons. HNCF before compared to HFNC after the test showed no statistical difference in paired *t* tests

We then removed the surgical mask and observed that SpO_2_ fell back to its initial value with a mean SpO_2_ of 91% (± 1) (Fig. 2). No patients revealed any complaints of discomfort and our observation confirmed that everyone accepted the surgical mask on the HFNC device very well.

## Discussion

All patients exhibited a hypoxemic acute respiratory failure related to Coronavirus Disease 2019 and presented refractory hypoxemia under classical nasal supplemental oxygen. As recommended, they benefited from a HFNC device to obtain a SpO_2_ above 90% [5]. As recommended, benefited of an HFNC device to obtain a SpO_2_ above 90% [5]. In this study, we demonstrated that the simple addition of a surgical mask on the patient’s face increased significantly the oxygenation of these hypoxemic COVID-19 patients admitted in ICU.

All started with the observation that these hypoxemic patients increased their SpO_2_ directly after receiving the surgical mask over the HFNC, a request of our hospital hygiene department. Adding this mask on the patient’s face while receiving HFNC oxygenation increases all the oxygenation parameters compared to classical HFNC therapy, without clinically significant change of PaCO_2_. All the settings of the HFNC devices were kept constant during the experiment.

We did not give to our patients any specific recommendation concerning mouth opening or closing during the experimentation, but we observed often an opened mouth with an increased room intake. The observed improvement in oxygenation parameters could be explained not only by an increased oxygen concentration in front of the mask but also by a decrease of room air entrainment that is known to dilute the gas mixture with less inspired O_2_ concentration [9]. The mask would then play a filter role by increasing the positive effect of the HFNC device and by decreasing the negative effect of entrainment of room air. We confirmed the additional effect of surgical mask to HFNC device rather than a favorable spontaneous evolution as its removal directly induced a return to previous oxygenation parameters as measured by the SpO_2_. Interestingly, none of our patients presented subjective complaints of discomfort by adding this surgical mask on top of HFNC.

The HFNC oxygen therapy is a well-known technique allowing heated and humidified gas with a maximum flow rate of 70 L/min and an adjustable oxygen fraction (FiO_2_) from 21 to 100% [10]. A recent meta-analysis showed that patients admitted with an acute hypoxemic respiratory failure from diverse aetiologies could improve oxygenation with HFNC compared to conventional oxygen therapy evolving towards a reduced need for tracheal intubation [11]. Likewise, it was recently demonstrated that the addition of a double-trunk mask on HFNC improves oxygenation in acute respiratory failure patients [12]. Non-invasive respiratory support plays an essential role in the treatment of COVID-19 patient with acute respiratory failure without the need of an urgent endotracheal intubation even if HFNC has not been assessed much yet. However, in adult patients admitted in ICU for an acute hypoxemic respiratory failure despite conventional oxygen therapy, as mentioned above, the Surviving Sepsis Campaign COVID-19 suggests the use of HFNC over Non-invasive positive-pressure ventilation (NIPPV) [5]. Recent study even observed an HFNC-positive response in moderate hypoxemic patients while failure rate increased as long as the PaO_2_/FiO_2_ decreased [13].

Importantly, this study was designed to assess the efficacy of adding a surgical mask on HFNC device and not to prevent endotracheal intubation.

Reducing the breath dispersion distance and aerosol generation during high-flow nasal ventilation to prevent SARS-CoV-2 transmission is a major issue. However, in vitro data using lung model with smoke generator or manikin are rather reassuring on this point. By using the same study method and similar breathing patterns, in vitro studies suggested that droplet dispersal during HFNC therapy was limited to the proximal space of the face and the cannula with even less dispersal distance of exhaled smoke compared to traditional high-flow oxygen therapy systems including non-rebreathing or Venturi masks that are traditionally used in acute hypoxemic respiratory failure [14, 15]. By studying different manikin models, in vitro and clinical studies, a recent review reported scientific evidences that use of HFNC during this pandemia has probably not increased either dispersion or microbiological contamination into the environment than other oxygen devices [16]. Furthermore, clinical studies evaluating bacterial environmental contamination of patients admitted in ICU for bacterial pneumonia and treated either by HFNC device or by conventional oxygen mask did not find any significant difference in bacterial counts in air or contact surface [17]. These data support the fact that there is actually no scientific proof of an increased bio-aerosols dispersion through an HFNC device compared to conventional high-flow oxygen therapy. Moreover, computational fluid dynamic simulations reported that wearing a surgical mask over HFNC might reduce aerosol droplets dispersion [18].

Our study presents several limitations. We enrolled a limited number of patients as we only included them in the presence of the investigators. We focused essentially in improving the oxygenation parameters. In this regard, specific measurements, such as minute ventilation, were not performed. Also, we did not enroll severe COPD patients and these data might not be generalizable to this population. However, PaCO_2_ measurement did not show any clinically significant increase, and it is worth noting that HFNC is proposed to treat those patients at home [19]. We further believe that targeting an SpO_2_ of 90% would limit the risk of oxygen-induced hypercapnia. Finally, the exact FiO_2_ delivered by the system while using the face mask was not measured, as already proposed by other authors [8].

## Conclusion

Our study suggests that a surgical mask placed on patient’s face already treated by a High-flow nasal cannula device would offer an advantage in terms of oxygenation in COVID-19 patients admitted in ICU with severe hypoxemic respiratory failure. Moreover, this oxygenation improvement is associated with neither a clinically significant change in the PaCO_2_ nor subjective patient complaints.

## Data Availability

All data generated or analysed during this study are included in this published article.

## Abbreviations

SARS-CoV-2: Severe acute respiratory syndrome coronavirus 2
COVID-19: Coronavirus disease 2019
ICU: Intensive care unit
HFNC: High-flow nasal cannula
NIPPV: Non-invasive positive-pressure ventilation
